# Relation of sensory processing and stomatognical system of oral respiratory children

**DOI:** 10.1590/2317-1782/20212020251

**Published:** 2021-10-25

**Authors:** Ana Carollyne Dantas de Lima, Raquel Costa Albuquerque, Daniele Andrade da Cunha, Camilla Albertina Dantas de Lima, Sandro Júnior Henrique Lima, Hilton Justino da Silva

**Affiliations:** 1 Departamento de Terapia Ocupacional, Universidade Federal da Paraíba – UFPB - João Pessoa (PB), Brasil.; 2 Departamento de Terapia Ocupacional, Universidade Federal de Pernambuco – UFPE - Recife (PE), Brasil.; 3 Departamento de Fonoaudiologia, Universidade Federal de Pernambuco – UFPE - Recife (PE), Brasil.; 4 Departamento de Oceanografia, Universidade Federal de Pernambuco – UFPE - Recife (PE), Brasil.; 5 Hospital Barão de Lucena – HBL - Recife (PE), Brasil.

**Keywords:** Stomatognathic System, Mouth Breathing, Sensation, Sensation Disorders, Sensory Deprivation

## Abstract

**Purpose:**

To verify the relationship between sensory processing and changes in the functions of the stomatognathic system in mouth breathing children, characterizing their sensory processing and comparing it with that of nasal breathing children.

**Methods:**

50 children (5 to 12 years) who were diagnosed with mouth breathing and 50 without signs and symptoms of mouth breathing or allergic rhinitis were selected to be part of the control group, matched for age and sex. Oral and nasal breathing children underwent sensory processing evaluation, through the Sensory Processing Measure – home form, and mouth breathers, through the evaluation of orofacial motricity through the Orofacial Myofunctional Evaluation with score. The results were presented in table form and with their respective absolute and relative frequencies.

**Results:**

Most of the children evaluated were male, with an average age of eight years. Most mouth breathers presented alteration in the processing of all senses, with a statistically significant relationship when compared to nasal breathers. There was a relationship, in mouth breathers, between proprioceptive sensory processing and the movement of the cheeks, visual sensory processing and head movement during swallowing, and between the type of chewing and tactile sensory processing.

**Conclusion:**

After analyzing the data, it was possible to see that the sensory processing of all systems presents with changes in mouth breathers and that this poor processing is related to orofacial mobility, as well as functions of the stomatognathic system, in addition to the type of chewing of this population.

## INTRODUCTION

The stomatognathic system is composed of structures related to vital functions (breathing, sucking, chewing and swallowing) and social functions (phonation and articulation) directly interconnected and related to survival. In this sense, changes in any of them can lead to a general imbalance in this system, leading to difficulties in daily life and, consequently, in quality of life^(1,2)^.

Breathing, one of the most important of these functions, occurs physiologically through the nasal route, protecting the upper airways, ensuring the proper development of structures, and functioning of the craniofacial complex. However, changes in breathing mode are common, especially in children, leading to mouth breathing. The causes of changes in breathing mode can be classified as obstructive (septal deviation, presence of a foreign body, mucosal hyperplasia, hyperplasia of the pharyngeal or palatine tonsils) and non-obstructive (sagging of phono articulatory organs and/or habitual functional mouth breathing). Thus, these changes can impede the passage of air through the nostrils, making the individual breathe through the mouth^(3-5)^.

The altered breathing mode leads to greater exposure of the upper airways, leading to inadequate development of the craniofacial complex, associated with abnormal functions of chewing, swallowing, tongue and lip posture^(6-8)^. In addition to breathing problems, chewing, swallowing, posture and tonicity of speech organs, mouth breathers may also present alterations in speech, voice, and body posture, which influence their performance of activities^(1,2,9)^. There is also evidence of alterations in smell, taste and auditory system, described as sensory dysfunctions^(3,10-14)^. Although studies suggest these dysfunctions, the characterization of other sensory systems, as well as the description of the sensory processing of oral breathers and the implications of these factors in daily life are not yet reported in the literature, and this study is original concerning the relationship between sensory processing and the stomatognathic system of oral breathers.

Sensory processing refers to a neurological function responsible for filtering, interpreting, organizing and modulating information received from the environment and from the body itself through the senses, favoring the selection of relevant information for an adequate response, allowing the performance of daily activities. Thus, initially, the sensory record occurs, where information is received by the environment and transduced into electrochemical stimuli for neuronal conduction. After that, sensory modulation occurs, where the physical characteristics of the stimulus are analyzed in terms of intensity, frequency, duration and specificity. Then, the stimulus is discriminated from perceptive analysis, regarding spatial and temporal qualities and, finally, there is the process of planning and organizing the behavior, which corresponds to the ideation, planning and execution of a motor action, executive function of the central nervous system known as praxis^(15-17)^.

In view of the problems exposed, children with mouth breathing can show a decline in daily life, educational and leisure activities caused by respiratory, motor and sensory impairments generating reduction in the level of functionality, due to agitation, inattention, sleep disorders, difficulty in performing activities that require physical effort and postural change according to progression of the condition^(3,18)^. Therefore, considering that childhood is an important stage of life for the individual's cognitive, motor and social development, any change in the performance of their activities can lead to consequences in the formation of their occupational role, directly interfering with their quality of life^(19)^.

Given the above, it can be hypothesized that the sensory processing of mouth breathing children presents dysfunction when compared to nasal breathing children, which can favor imbalance (or generate changes) in the performance of stomatognathic functions. Thus, both dysfunctional sensory processing and the stomatognathic system with imbalance and alteration can influence the performance of daily activities such as eating, leisure, school activities, rest and sleep.

Thus, this study aims to verify the relationship between sensory processing and changes in the functions of the stomatognathic system in mouth breathing children, characterizing their sensory processing and comparing it with that of nasal breathing children. Still, it aims to present the possibility of analysis and multi professional clinical intervention, contributing to the differential diagnosis and specialized intervention directed to each case.

## METHODS

This is an observational, analytical and cross-sectional study, with a random population sample, of convenience and obtained by spontaneous demand, according to the eligibility criteria. The studied population consisted of a case group and a control group, matched for age and sex. The case group (OB) was formed by children with signs and symptoms of mouth breathing (data obtained through the application of the protocol of signs and symptoms of mouth breathing)^(20,21)^, randomly recruited from among the children seen at the *Ambulatório de Alergia e Alergologia* at the *Hospital das Clínicas da Universidade Federal de Pernambuco*, during the period of data collection. The control group (NB) was formed by nasal breathing children without a diagnosis of allergic rhinitis (information obtained by consulting the medical record) and without signs and symptoms of mouth breathing (data obtained by applying the protocol of signs and symptoms of mouth breathing). They were in good health, randomly recruited from the children attended at the *Clínica escola de Odontologia* of the *Universidade Federal de Pernambuco*. Children aged between five and 12 years were selected, and children who presented genetic syndromes were excluded from both groups; orofacial malformations; use of orthodontic braces; intellectual disability (which hindered communication and response to tests); neurological disorders; already diagnosed sensory processing disorder; diagnosed visual and hearing alterations and those who were undergoing speech therapy. We obtained eligibility criteria by consulting the medical records available at the services and interviewing parents or guardians.

### Instruments

Data corresponding to biological, socioeconomic, environmental variables and some eligibility criteria (visual and auditory alterations, sensory processing already diagnosed and speech therapy in progress) were obtained by applying a form prepared for research with the child's parents or guardians. It contained questions about the sociodemographic data of the child and his mother (age, gender, education and residence) as well as data on the child's health (type of breastfeeding and feeding; sleep habits) and family income.

The classification of the child as mouth breathing was performed by a single speech therapist in the area of ​​orofacial motricity through a protocol of signs and symptoms of mouth breathing. An interdisciplinary team formed by speech therapists, dentists and otolaryngologists prepared this protocol with the objective of offering speech therapy diagnosis of mouth breathing. It was based on studies by Genaro et al.^(20)^ and used for classification in the study by Melo et al.^(21)^. This protocol is composed of three parts: information about the breathing mode, symptoms related to the breathing mode reported by the patient (both with questions that must be answered by the companion or by the patient when they are over 18 years old) and signs related to the breathing mode, observed on the valuation date.

The evaluation of sensory processing was performed by a single occupational therapist, using the Sensory Processing Measure – Home form (SPM). The SPM consists of 75 items and must be answered by a parent or primary caregiver of the child. It presents eight standardized and normatively referenced results: Social Participation (SOC), Vision (VIS), Hearing (HEA), Touch (TOU), Body Knowledge (BOD), Balance and Movement (BAL), Idea Planning (PLA) and Total Sensory Systems (TSS). This scale assesses the sensory processing, praxis and social participation of children between 5 and 12 years old who are attending school. The questions that make up the assessment are related to the behavior that the child presents in certain situations, reacting to sensory stimuli presented. Thus, parents answer how often the child presents a certain behavior in face of sensory stimulation, in a Likert scale in which the options are never, occasionally, frequently and always (scoring from 1 to 4 respectively). At the end, the scores of each scale (SOC; VIS; HEA; TOU; BOD; BAL; PLA and TSS) are added individually and placed in a table for matching T scores. The scores range from 40 to 80T and the score of each scale classifies the functioning of sensory processing into three types of interpretation: typical, some problem or definitive dysfunction, related to each sensory system and the final sum of all evaluated systems. Thus, the higher the child's score (the higher his T score), the greater the change in his sensory processing. For the present study, we used only the scales corresponding to the sensory systems (visual (VIS), auditory (HEA), tactile (TOU), proprioceptive (BOD) and vestibular (BAL)) and total systems (TSS). The results “some problem” and “definite dysfunction” were categorized as “dysfunction” since any change in one of the systems is already characterized as sensory processing problem^(22)^.

The assessment of orofacial motricity was performed using the protocol “Orofacial Myofunctional Assessment with Scores” (AMIOFE). All assessments were recorded for later appointment by the same speech therapist who performed the assessment of the oral breathing signs and symptoms protocol. This instrument is aimed at orofacial myofunctional assessment and is divided into aspects such as appearance and postural condition/position (lips, jaws, cheeks, face, tongue and palate); mobility (lips, tongue, jaw and cheeks); functions (chewing, swallowing and breathing) and other behaviors and signs of alteration (movement of the head or other parts of the body, altered posture and food escape). Each of these aspects is evaluated through observation and scored in scores, according to Felício and Ferreira^(23)^.

### Procedure

Initially, we selected the population of the case group (OB) by analyzing medical records provided by the service where the assessments were being carried out (*Ambulatório de Alergia e Alergologia*, *Hospital das Clínicas*, *Universidade Federal de Pernambuco*). After selecting the children from the medical records, using the eligibility criteria, on the day of the appointment scheduled by the service, the research procedures were described to the parents or guardians of the child for further consent. Upon acceptance to participate, after everyone responsible involved had signed the Informed Consent Term, the children were referred to perform diagnosis of oral breathing, through the protocol for identifying signs and symptoms of oral breathing. After identification, an interview was carried out and the data registration form was filled out to collect sociodemographic data and some eligibility criteria. After this screening, in case there was no feasibility, the evaluations were scheduled for the date of the child's next visit to the service.

When it was possible to carry out the evaluation right after the screening, the sensory processing evaluation instruments were applied, in the form of an interview, through the SPM – Home Form, and the orofacial motricity, through the AMIOFE instrument. The assessment protocols were applied in the presence of the parents or guardians of the child, in a room designed for this purpose, in the clinic where the data collection was being carried out.

After collecting the population of the case group (OB), the control group (NB) was selected by pairing age and sex. We should clarify that the participants of the control group were not collected at the same place and time as the case group. Since the selection was made by pairing age and gender, it was necessary to complete the collection of the case group for subsequent selection of the control group. After pairing, eligibility criteria were collected through analysis of medical records provided by the *Clínica escola de Odontologia da Universidade Federal de Pernambuco* and an initial interview with the child's parents or guardians. After the screening, upon acceptance of participation in the research, we referred the children to carry out diagnosis of oral breathing, through the protocol of identification of signs and symptoms of oral breathing. When the result was nasal breathing, the sensory processing was evaluated through the SPM – Home Form.

For the assessment of orofacial motricity, the participants of the case group (OB) remained in a chair with a back, in an upright position; with feet supported, upper and lower limbs relaxed and uncrossed; hands on thighs, mandible parallel to the ground; with the head unsupported. Choosing this posture provides more comfort and spontaneity to the head and neck. To assess swallowing and chewing, the participants were instructed to drink a glass of 180 ml of mineral water (un-carbonated) and a piece French bread (25 g) that was weighed by a JL-3 Precision scale (500 mg capacity), as usual, respectively^(23)^. The entire application of this protocol was monitored and guided by a speech therapist and filmed with the consent of parents or guardians for further analysis. The application of the PMS was performed by an occupational therapist through an interview with the children's parents or guardians.

### Data analysis

For data analysis, we used SPSS software (Statistical Package for the Social Sciences) in version 18 and Excel 2010^®^. The results were presented in table form and with their respective absolute and relative frequencies. To verify the existence of an association between sensory processing and the clinical characteristics studied, the Chi-Square Test or Fisher's Exact Test was used, when necessary. All tests were applied with a 95% confidence interval and p values ​​< 0.05 were considered statistically significant.

This project was submitted to the ethics committee for research with human beings of the Health Sciences Center of the *Universidade Federal de Pernambuco*, in accordance with resolution 466/12 of the National Health Council of the Ministry of Health of Brazil regarding research with human beings. The same was accepted through opinion nº. 992.769, CAAE: 42103315.3.0000.5208. The evaluations were only carried out after everyone responsible involved had signed the Informed consent form.

## RESULTS

One hundred children participated in the study, 50 mouth breathers (OB) and 50 nose breathers (NB), 68 males and 32 females, with a mean age of eight (± 2.15) years and attending elementary school I (64% OB; 84% NB). Most mothers (82%) had a mean age of 35.2 (± 7.2) years and had completed high school (54.0% OB; 56% NB).

By analyzing sensory processing (Table 1), we found that 41 (68.0%) mouth-breathing children had sensory processing dysfunction. The sensory systems that presented the highest frequency of dysfunction in this population were the visual (65.0%), the tactile (touch) (70.0%), and the vestibular (balance and movement) (78.0%) systems. When compared to the sensory processing of nasal breathing children, the analysis of all systems showed statistical significance, with more attention to the total score (total sensory systems) and the vestibular system (balance and movement), both with a value of p = 0.000.

**Table 1:** Frequency distribution of sensory processing data for the case (OB) and control (NB) groups. Recife - PE, 2017

**Case/Healthy control Group**	Sensory System **(Total Sensory System)**			**p** ^*^	**OR**	**CI (95%)**
	Typical N (%)	Dysfunction N (%)		<10^-4^	0.13	0.05-0,34
OB	9 (22%)	41 (68%)	
NB	31 (78%)	19 (32%)	
Total	40 (100%)	60 (100%)	
**Case/Healthy control Group**	Sensory System **(Visual)**			p*	**OR**	**CI (95%)**
	Typical N (%)	Dysfunction N (%)		0.004	0.31	0.14-0.71
OB	20 (37%)	30 (65%)				
NB	34 (63%)	16 (35%)				
Total	54 (100%)	46 (100%)				
**Case/Healthy control Group**	Sensory System **(Auditory)**			p*	**OR**	**CI (95%)**
	Typical N (%)	Dysfunction N (%)		0.028	0.40	0.17-0.95
OB	28 (65%)	22 (42%)				
NB	38 (58%)	12 (35%)				
Total	66 (100%)	34 (100%)				
**Case/Healthy control Group**	Sensory System **(Proprioceptive)**			p*	**OR**	**CI (95%)**
	Typical N (%)	Dysfunction N (%)		0.008	0.34	0.15-0.77
OB	21 (38%)	29 (64%)				
NB	34 (62%)	16 (36%)				
Total	55 (100%)	45 (100%)				
**Case/Healthy control Group**	Sensory System **(Tactile)**			p*	**OR**	**CI (95%)**
	Typical N (%)	Dysfunction N (%)		0.001	0.23	0.10-0.55
OB	20 (35%)	30 (70%)				
NB	37 (65%)	13 (30%)				
Total	57 (100%)	43 (100%)				
**Case/Healthy control Group**	Sensory System **(Vestibular)**			p*	**OR**	**CI (95%)**
	Typical N (%)	Dysfunction N (%)		<10^-4^	0.14	0.060-0.37
OB	20 (33%)	30 (78%)				
NB	41 (67%)	9 (22%)				
Total	61 (100%)	39 (100%)				

Caption: OR = Odds ratio; CI = Confidence Interval; p*>0.05

Regarding the myofunctional aspects of mobility, most OB children did not show changes in lip, tongue, jaw and cheek movements (Table 2). In the functions of the stomatognathic system (swallowing and chewing), the presence of head movement, facial muscles tension and food escape were noticed for the majority of the studied population (Table 2). Within the chewing function, the most frequent type of chewing was alternated bilateral (42.2%), followed by preferential unilateral (40.0%) and chronic unilateral (18.0%).

**Table 2:** Frequency of orofacial myofunctional aspects of labial, tongue, jaw and cheek mobility and swallowing and Chewing functions of the case group (OB), Recife-PE, 2017

**Variable (Mobility)**	**Normal**		**Changed**	
	*F*	%	*f*	%
Lip Movements	31	62.0	19	38.0
Tongue Movements	39	78.0	11	22.0
Jaw Movements	36	72.0	14	28.0
Cheeks Movements	41	82.0	09	18.0
**Variable (Deglutition)**	**Absent**		**Present**	
	*F*	**%**	*f*	**%**
Head Movement	19	38.0	31	62.0
Facial Muscle Tension	23	46.0	27	54.0
Food Escape	19	38.0	31	62.0
**Variable (Chewing)**	**Absent**		**Present**	
	*F*	**%**	*f*	**%**
Head Movement	20	40.0	30	60.0
Altered Posture	16	32.0	34	68.0
Food Escape	18	36.0	32	64.0

Regarding the relationship between sensory processing and the stomatognathic system, there was a statistically significant relationship between the proprioceptive sensory processing (body awareness) and the myofunctional aspect of cheek mobility (p = 0.040) and between visual processing and head movements during swallowing (p=0.042). Tactile sensory processing and the type of chewing were also statistically significant (p=0.03). It is possible to see, through binary logistic regression, that children with mouth breathing have a greater tendency to show changes in sensory processing and more specifically in vestibular sensory processing, that is, in balance and movement (Table 3).

**Table 3:** Binary Logistic Regression showing the influence of the combination of all oral breathing factors on the sensory processing of children, Recife-PE, 2017

**Sistema**	**B**	**p**	**OR**	**CI 95%**
TSS	1.61	**0.030** ^*^	4.999	1.16-21.45
Visual	0.215	0.709	1.239	0.40-3.82
Auditory	-0.569	0.365	.566	0.16-1.94
Tactile	0.343	0.577	1.410	0.42-4.71
Proprioceptive	-0.315	0.609	.730	0.22-2.44
Vestibular	1.594	0.005*	4.923	1.61-15.07

Caption: TSS = Total Sensory System; B = Coefficient B; OR = Odds ratio; CI = Confidence Interval; p*>0.05

## DISCUSSION

After analyzing the data, it was clear that the sensory processing of all systems presents changes in mouth breathers and that this poor processing is related to orofacial mobility, as well as to the functions of swallowing and chewing.

The population studied was similar to that of other studies that evaluated mouth-breathing children, mostly male and with a mean age of eight years, enrolled in elementary school^(8,11,13,21,24)^. Note that the age group selected in this study was limited to the specificity of the sensory processing assessment instrument, which contains questions related to the age group from five to 12 years old. It is also important to emphasize that the population of nasal breathers was paired by sex and age with the population of mouth breathers, avoiding any specificity regarding these variables. Maternal age and guardians' education level also favored responses to the instruments, as some of them depended on interpretation and responses within the Likert scale.

Contrary to what we observed in mouth-breathing children, we detected no alterations in the sensory processing of nasal breathing children. This makes clear the influence of mouth breathing in this processing, as this condition does not allow for adequate sensory input, making it difficult to record sensations and possibly changing the entire processing sequence, leading to an inadequate adaptive response to the environment and difficulty in the performance of everyday activities. It is important to emphasize that sensory systems work in an integrated way for the optimal performance of human actions, thus, each one of them depends on the proper functioning of others so that adaptive responses to environmental demands are adequate^(25)^.

The alterations found in the visual, vestibular and proprioceptive systems in the population of mouth breathers studied can be explained by the head, neck and shoulder posture common to mouth breathers. Changing the position of the head and shoulders requires the mouth breather to lean the body forward, move the arms back, and the feet are in inversion, to achieve balance^(26)^. The harmony of the body in relation to its segments and the environment that surrounds it depends on the integration of information from the visual, somatosensory and vestibular systems, maintaining an efficient body balance and adequate posture^(25)^. Therefore, dysfunction of any of these systems can affect posture control and balance. Thus, the results obtained are in agreement with the existing literature, suggesting the relationship between the proprioceptive and vestibular sensory processing and the oral breathing pattern of children are due to changes in posture.

In a literature review, Machado et al.^(26)^ presented articles that related body posture with the functions of the stomatognathic system, postulating that changes in stomatognathic functions, especially breathing, can contribute to deficiencies in alignment and postural control, due to the relationship between the trigeminal system and the nervous structures involved in posture control. In an observational study with a control group, Conti and Collaborators^(6)^ evaluated 306 mouth breathers (OB) and 124 nose breathers (NB), observing a moderate postural change in 60.74% in the OB population, with a statistically significant difference (p ≤ 0 .0002) when comparing the position of the head, shoulders, feet and plantar arch segments of the case group with the control group (NB). Roggia et al.^(9)^ evaluated the vestibular, visual and somatosensory systems in an integrated way, showing the influence of the three systems on the balance and posture of mouth breathing children. The authors concluded that mouth breathers showed more postural changes than the control group (nasal breathers) and related the difficulties encountered with the structural and physiological changes caused in the sensory systems.

In this sense, the responses of visual and tactile processing, presented by mouth breathers in this study, in addition to being related to the postural aspects presented by this population, can also be justified by other factors. Regarding visual processing, the evaluation used (SPM – home form) obtains the results through questions related to the responses that individuals offer to a sensory stimulus. Thus, most of the questions in the session that assesses the visual system are focused on attention. Some studies report that mouth breathers present attention and concentration problems, due to respiratory demands that affect, among other factors, rest and sleep, making this population more sleepy during the day and, consequently, more inattentive^(12,14)^.

The tactile system is directly related to the proprioceptive and gustatory system. The latter is directly influenced by the olfactory system and consequently by breathing. Thus, the mouth breathers in this study, due to the decrease in smell, taste and tone of the orofacial muscles and oral malocclusion (open or cross bite and absence of anterior dental elements), may have selected the food for its consistency and consequently ease of ingestion, choosing to consume soft and liquid foods^(13,26)^. This dietary restriction reduces the possibility of tactile and possibly bodily oral sensory experiences, leading to the absence of adequate sensory experimentation, poor recording of information and poor sensory processing.

From the point of view of sensory integration, the proprioceptive, vestibular and tactile senses are emphasized for being primitive and primary, dominating the interaction of children with the world in the first years of life^(10)^. Thus, due to the assumed body postures and consequently the vestibular, tactile, proprioceptive and visual processing alterations, the evaluated mouth-breathing children may have presented characteristics of disorganized motor functions, decreased muscle tone and impaired balance. These functions are related to praxis, a skill that depends on the proper functioning and integration of sensory systems, especially tactile, proprioceptive and vestibular, to conceptualize, plan and execute an unusual motor act. Thus, changes in these systems can lead to difficulty in performing motor activities, such as the transition from one body position to another and sequenced or timed actions involved in a task. These characteristics hinder the performance of children in daily activities such as eating, dressing, writing and school activities, representing a significant performance deficit that can lead to problems in social participation, considering this stage of the child's development, where training is being initiated, as well as group identification^(10,18,19)^.

With regard to the auditory system, more mouth breathers with typical sensory processing were observed (56%). However, when compared to the control group (76% of typical processing) it is possible to observe a significant difference, which reflects an influence of mouth breathing in the processing of this information, since the children were matched for age and gender, and are in similar educational process. One of the consequences of oral breathing, due to the malfunction of the Eustachian tube, is the development of otitis media and, consequently, of mutable hearing^(11,27)^. Therefore, mouth breathing is a condition that may be associated with a failure in auditory processing, with otitis and hearing fluctuation being possible causes of this problem. In school-age children, as in the case of this research (five – 12 years), the effects of mouth breathing on the auditory system deserve special attention, since it is then that there is the greatest development of human hearing skills and, therefore, fluctuations in hearing must be observed for the normal development of this system to occur, avoiding learning difficulties^(28)^.

In a study with a similar population, Correa et al.^(27)^ found significant results between the case (mouth breathers) and control (nasal breathers) groups for conditions of competitive left and right ears, alteration related to auditory organization and integration, auditory closure and temporal pattern, concluding that mouth breathing children have lower performance in auditory processing skills than nose breathing children. Note that the evaluation of sensory processing carried out in this study observes the adaptive behavioral responses to received stimuli, evaluating how children react most of the time to the demands of the environment. This analysis differs from most studies related to auditory processing, which focus on the quantification of electrical impulses, as well as the presence or absence of auditory and speech responses^(11,27,29)^. Regardless of the cause, changes in auditory sensory processing can influence the performance of these children, especially in school activities. As this dysfunction can lead to difficulties in language development, attention deficits, graphic and reading errors, slowness, difficulty following oral instructions and selecting auditory stimuli in noisy environments, agitation, hyperactivity or apathy and alterations in the notion of laterality^(24,27,28)^.

In relation to the stomatognathic system, alterations in the mobility of the lips, tongue, jaw and cheeks were not found in the majority of the population of mouth-breathers. However, in the functions of swallowing and chewing, the presence of head movement, facial muscles tension and food escape were observed. These alterations show incapacity in these functions, probably due to a reduction in the contraction of some muscles, contraction of per orbicular muscles and flaccidity of phono articulatory organs. These characteristics were also present in studies that compared oral and nasal breathing children^(13,26)^.

For Machado et al.^(26)^, the OB, in many cases, cannot chew food correctly due to the need to breathe, because when opening the mouth to do so, there are adaptations and imbalance of orofacial structures and functions that compromise chewing and swallowing, and, consequently, it generates eating difficulties. The mouth-breathing child must keep the passage of air through the mouth free in order to be able to breathe. Thus, when ingesting food, it is necessary to swallow it quickly to release the air passage through the mouth to breathe again^(13)^. In this sense, the literature shows that the chewing time of OB is faster than that of the individual who breathes through the nose^(13,26,29)^, which may explain the alterations found in the functions of chewing and swallowing.

As for the type of chewing, most of them had alternated bilateral chewing (42%), a pattern that is considered ideal and responsible for the existence of an orofacial balance. This result was similar to the study by Silva et al.^(29)^, which identified, in a smaller sample, the prevalence of alternating bilateral chewing in 20 of the 23 OB children evaluated. This can be explained by the fact that oral breathing can change the structures of the stomatognathic system depending on the degree of severity of the nasal obstruction and the time of interference, this assessment being subjective because it is a population still in development. Despite this, there was an approximation of the percentage of OB with preferential unilateral chewing (40%), which brings the results closer to those of a study carried out with a similar population, in the same age group^(13,29)^.

When sensory processing was related to the structures and functions of the stomatognathic system of mouth breathers, it was possible to observe a statistically significant response between proprioceptive processing and cheek movement as well as visual processing and head movement during swallowing and the relationship between the type of chewing and tactile processing. These data show the possible influence of oral breathing on the poor processing of sensory information and the consequences that this can bring to the stomatognathic functions. With regard to proprioceptive processing and cheek movement, it is possible to observe that despite the proprioceptive dysfunction, most mouth breathers had normal cheek movement, which can be explained by the type of material (bread and water) that was used to assess chewing and swallowing. These supplies probably do not require proprioceptive inputs different from the usual ones for this population, so even with poor proprioceptive processing the movement did not change, as it no longer required this system.

The responses obtained by the relationship between visual processing and head movement during swallowing show that the greatest dysfunction in visual processing occurred when there was head movement during swallowing. It is known that the mouth breather assumes a head compensation posture to keep the bi-pupillary plane of the horizon line parallel to the ground to maintain balance. This occurs through a slight backward tilt of the head^(8,9)^. This fact may justify the difficulty in visual maintenance during swallowing and the presence of head movement in this function. Another point is the large amount of sensory stimuli present in the feeding activity and in the swallowing function, which may require a greater division of attention from the stimuli, a factor that is impaired in mouth breathing children, especially in relation to visual processing.

Regarding the relationship between the chewing type and tactile processing, it appears that when processing is typical, chewing is alternated, whereas when there is processing dysfunction, the preference is chewing. These data show the influence that tactile processing (mainly sensory input) can exert on mastication and the masticatory type. Knowing that mouth breathers have food selectivity, mainly related to food texture and consistency, the lack of stimulation and exploration in this region justifies the tactile dysfunction presented and the type of chewing preference. Chewing undergoes modifications until the stomatognathic system matures and dentition is fully developed. From then onwards, it is perfected and, at the same time, there is a morphological adaptation of the occlusal surface. The masticatory muscles play the most important role in the process, although the tongue and facial muscles also participate^(13,29)^. Therefore, the consistency of the food directly influences mastication, adapting it to the type of food ingested, modifying the intensity of strength, pressure and the number of chewing strokes, stimulating and working the masticatory muscles. Thus, the consistency of the food will influence both the type of tactile stimulus provided and the preference of the chewing side.

Although no statistically significant relationship was found between other sensory systems and stomatognathic functions, it is clear that that there is a tendency for the presence of head movement, facial muscles tension and food escape during mastication and swallowing when processing sensory is dysfunctional. These data show that the changes presented in the chewing and swallowing functions of the evaluated mouth breathers may be influenced by the change in sensory processing, as this must occur in an integrated way to ensure adequate performance in daily activities^(25)^. When there is any change in one of these systems, there is a negative feedback relationship with the other senses, causing deficits in the performance of activities.

Due to the objective of presenting the absence or presence of alteration in the sensory processing of oral breathers, some inferences could not be made in this study, as they would require a classification of the type of alteration in the sensory processing of each system. Another point to be considered was that the study was done with the population of only one city in Brazil, which may have influenced the answers given, especially those from the SPM Home Form, which, as an instrument that assesses adaptive responses to the environment, is influenced by interpretation and population culture. Thus, we suggest studies that assess populations from different Brazilian states and regions, as well as those characterizing the type of sensory processing disorder of each system evaluated, thus ensuring the possibility of inferences about the number of stimuli offered and the direction of clinical practices.

## CONCLUSION

Thus, the findings showed that the sensory processing of mouth breathing children is altered for all evaluated systems, especially when compared with data from nasal breathing children. It also shows that there was a significant relationship between the proprioceptive, visual and tactile sensory processing with the movement of the cheeks and head during swallowing, as well as with the type of chewing performed by mouth-breathers.

Because it only presents the presence or absence of changes in sensory processing, this study suggests deepening the clinical observations of this population to characterize the type of sensory processing disorder in each system and ensure a targeted and individualized intervention, considering the personal characteristics of activities performed and life contexts. Finally, these observations provide the basis for a broader look at the population of mouth breathers, with regard to the interaction of sensory systems with this health condition and better decision-making for multidisciplinary interventions, including occupational therapists in the assessment and intervention through sensory integration therapy, the type of treatment performed only by this professional.
