# Supplementary material for: Relation of sensory processing and stomatognical system of oral respiratory children
Source: Codas. 2021 Oct 25;34(2):e20200251. doi: 10.1590/2317-1782/20212020251 (PMC9769425; doi:10.1590/2317-1782/20212020251)
Supplement: Translation

# Translation: Relação do processamento sensorial e sistema estomatognático de crianças respiradoras orais

**Authors:** Ana Carollyne Dantas de Lima, Raquel Costa Albuquerque, Daniele Andrade da Cunha, Camilla Albertina Dantas de Lima, Sandro Júnior Henrique Lima, Hilton Justino da Silva

**Affiliations:** 1 Departamento de Terapia Ocupacional, Universidade Federal da Paraíba – UFPB - João Pessoa (PB), Brasil.; 2 Departamento de Terapia Ocupacional, Universidade Federal de Pernambuco – UFPE - Recife (PE), Brasil.; 3 Departamento de Fonoaudiologia, Universidade Federal de Pernambuco – UFPE - Recife (PE), Brasil.; 4 Departamento de Oceanografia, Universidade Federal de Pernambuco – UFPE - Recife (PE), Brasil.; 5 Hospital Barão de Lucena – HBL - Recife (PE), Brasil.

**Keywords:** Sistema Estomatognático, Respirador Bucal, Sensações, Transtorno das Sensações, Privação Sensorial

## Abstract

**Objetivo:**

Verificar a relação entre o processamento sensorial e as alterações das funções do Sistema Estomatognático de crianças respiradoras orais, caracterizando o processamento sensorial destas e comparando-o com o de respiradoras nasais.

**Método:**

Foram selecionadas 50 crianças (5 a 12 anos) que apresentaram diagnóstico de respiração oral e 50 sem sinais e sintomas de respiração oral ou rinite alérgica para fazer parte do grupo controle, pareadas por idade e sexo. As crianças respiradoras orais e nasais passaram por avaliação do processamento sensorial, através da Sensory Processing Measure – Home form, e as respiradoras orais por avaliação da motricidade orofacial através da Avaliação Miofuncional Orofacial com Escore. Os resultados foram apresentados em forma de tabela e com suas respectivas frequências absoluta e relativa.

**Resultados:**

A maioria das crianças avaliadas foi do sexo masculino, estando com idade média de 8 anos. A maioria dos respiradores orais apresentou alteração no processamento de todos os sentidos, com relação estatisticamente significativa quando comparados com os respiradores nasais. Houve relação, nos respiradores orais, entre o processamento sensorial proprioceptivo e o movimento das bochechas, processamento sensorial visual e movimentação da cabeça durante a deglutição e entre o tipo de mastigação e o processamento sensorial tátil.

**Conclusão:**

Após análise dos dados foi possível perceber que o processamento sensorial de todos os sistemas se apresenta com alteração nos respiradores orais e que esse mau processamento se relaciona a mobilidade orofacial, bem como com funções do Sistema Estomatognático, além do tipo de mastigação dessa população.

## INTRODUÇÃO

O Sistema Estomatognático é composto por estruturas relacionadas as funções vitais (respiração, sucção, mastigação e deglutição) e sociais (fonação e articulação) diretamente interligadas e relacionadas a sobrevivência. Nesse sentindo, alterações em qualquer uma delas pode acarretar desequilíbrio geral desse sistema, levando a dificuldades na vida cotidiana e consequentemente na qualidade de vida^(1,2)^.

A respiração, uma das mais importantes dessas funções, ocorre de forma fisiológica por via nasal, protegendo as vias aéreas superiores e garantindo o desenvolvimento adequado das estruturas e funcionamento do complexo craniofacial. Porém, é comum ocorrer alterações no modo respiratório, principalmente em crianças, levando à respiração oral. As causas de alteração no modo respiratório podem ser classificadas em obstrutivas (desvio de septo, presença de corpo estranho, hiperplasias de mucosa, hiperplasias das tonsilas faríngeas ou palatinas) e não obstrutivas (flacidez dos órgãos fonoarticulatórios e/ ou respiração oral funcional, por hábito). Desta forma, essas alterações podem impedir a passagem de ar pelas narinas, fazendo com que o indivíduo respire pela boca^(3-5)^.

O modo respiratório alterado leva a maior exposição das vias aéreas superiores, acarretando o desenvolvimento inadequado do complexo crânio-facial, estando associado a funções anormais de mastigação, deglutição, postura da língua e lábios^(6-8)^. Além dos problemas de respiração, mastigação, deglutição, postura e tonicidade dos órgãos fonoarticulatórios, os respiradores orais podem apresentar também alterações na fala, voz, e postura corporal, que influenciam no desempenho de suas atividades^(1,2,9)^. Há evidências também da presença de alterações no olfato, paladar e sistema auditivo, descritas como disfunções sensoriais^(3,10-14)^. Apesar de estudos sugerirem essas disfunções, a caracterização dos demais sistemas sensoriais, bem como a descrição do processamento sensorial dos indivíduos respiradores orais e as implicações desses fatores no cotidiano ainda não são relatadas na literatura, sendo este estudo original no que se refere a relação entre o processamento sensorial e o Sistema Estomatognático de respiradores orais.

O processamento sensorial refere-se a uma função neurológica responsável por filtrar, interpretar, organizar e modular as informações recebidas do ambiente e do próprio corpo através dos sentidos, favorecendo a seleção das informações relevantes para uma resposta adequada, permitindo o desempenho das atividades cotidianas. Desta forma, inicialmente ocorre o registro sensorial, onde a informação é recebida pelo ambiente e transduzida em estímulos eletroquímicos para a condução neuronal. Após isto, ocorre a modulação sensorial, onde são analisadas as características físicas do estímulo quanto à intensidade, frequência, duração e especificidade. Em seguida, o estímulo é discriminado a partir de análises perceptivas, quanto as qualidades espaciais e temporais e, por fim, ocorre o processo de planejamento e organização do comportamento, que corresponde a ideação, planejamento e execução de uma ação motora, função executiva do Sistema Nervoso Central conhecida como praxia^(15-17)^.

Tendo em vista os problemas expostos, as crianças com respiração oral podem apresentar declínio nas atividades de vida diária, educacionais e de lazer ocasionado pelos comprometimentos respiratórios, motores e sensoriais que geram uma redução no nível de funcionalidade, por conta da agitação, desatenção, distúrbios do sono, dificuldade em realizar atividades que requerem esforço físico e alteração postural de acordo com a progressão do quadro^(3,18)^. Portanto, levando em consideração que a infância é uma fase da vida importante para a formação cognitiva, motora e social do indivíduo, qualquer alteração no desempenho das suas atividades pode levar a consequências na formação do seu papel ocupacional, interferindo diretamente na sua qualidade de vida^(19)^.

Diante do exposto, pode-se hipotetizar que o processamento sensorial de crianças respiradoras orais apresenta disfunção se comparado ao de crianças respiradoras nasais, o que pode favorecer ao desequilíbrio (ou gerar alterações) no desempenho das funções estomatognáticas. Assim, tanto o processamento sensorial disfuncional quanto o Sistema Estomatognático com desequilíbrio e alteração podem influenciar o desempenho de atividades cotidianas como alimentação, lazer, atividades escolares, descanso e sono.

Desta forma, este estudo tem como principal objetivo verificar a relação entre o processamento sensorial e as alterações das funções do Sistema Estomatognático de crianças respiradoras orais, caracterizando o processamento sensorial destas e comparando-o com o de crianças respiradoras nasais. Ainda, tem o objetivo de apresentar possibilidade de análise e intervenção clínica multiprofissional, contribuindo para o diagnóstico diferencial e a intervenção especializada e direcionada para cada caso.

## MÉTODO

Trata-se de um estudo observacional, analítico e transversal, com uma amostra populacional aleatória, de conveniência e obtida por demanda espontânea, segundo os critérios de elegibilidade. A população estudada foi composta por um grupo caso e um grupo controle, pareados por idade e sexo. O Grupo caso (RO) foi formado por crianças com sinais e sintomas de respiração oral (dado obtido através da aplicação do protocolo de sinais e sintomas da respiração oral)^(20,21)^, recrutadas de forma aleatória dentre as crianças atendidas no ambulatório de Alergia e Alergologia do Hospital das Clínicas da Universidade Federal de Pernambuco, durante o período da coleta de dados. O Grupo controle (RN) foi formado por crianças respiradoras nasais sem diagnóstico de rinite alérgica (informação obtida através de consulta ao prontuário) e sem sinais e sintomas de respiração oral (dado obtido através da aplicação do protocolo de sinais e sintomas da respiração oral), em boas condições de saúde, recrutadas de forma aleatória dentre as crianças atendidas na Clínica escola de Odontologia da Universidade Federal de Pernambuco. Foram selecionadas crianças com idade entre 5 e 12 anos, e excluídas, de ambos os grupos, crianças que apresentaram síndromes genéticas; malformações orofaciais; uso de aparelho ortodôntico; deficiência intelectual (que dificultasse a comunicação e resposta aos testes); distúrbios neurológicos; transtorno do processamento sensorial já diagnosticado; alterações visuais e auditivas diagnosticadas e que estivessem em atendimento fonoaudiológico. Os critérios de elegibilidade foram obtidos através de consulta aos prontuários disponibilizados nos serviços e entrevista com os pais ou responsáveis.

### Instrumentos

Os dados correspondentes às variáveis biológicas, socioeconômicas, ambientais e alguns critérios de elegibilidade (alterações visuais e auditivas, processamento sensorial já diagnosticado e tratamento fonoaudiológico em realização) foram obtidos através da aplicação de um formulário elaborado para pesquisa junto aos pais ou responsáveis pela criança. O mesmo continha perguntas quanto aos dados sociodemográficos da criança e sua mãe (idade, sexo, escolaridade e moradia) além de dados da saúde da criança (tipo de amamentação e alimentação; hábitos de sono) e renda familiar.

A classificação da criança como respiradora oral foi realizada por um único fonoaudiólogo da área de motricidade orofacial através do protocolo de sinais e sintomas da respiração oral. Este protocolo foi elaborado por uma equipe interdisciplinar formada por fonoaudiólogos, odontólogos e otorrinolaringologistas tendo como objetivo oferecer diagnóstico fonoaudiológico de respiração oral. Foi baseado nos estudos de Genaro et al.^(20)^ e utilizado para classificação no estudo de Melo et al.^(21)^. Esse protocolo é composto por três partes: informações sobre o modo respiratório, sintomas relacionados ao modo respiratório relatados pelo paciente (ambos com perguntas que deverão ser respondidas pelo acompanhante ou pelo paciente quando este for maior de 18 anos) e sinais relacionados ao modo respiratório, observados na data da avaliação.

A avaliação do processamento sensorial foi realizada por uma única terapeuta ocupacional, através da *Sensory Processing Measure – Home form (SPM)*. A SPM é constituída por 75 itens e tem de ser respondida por um dos pais ou cuidador principal da criança. Apresenta 8 resultados estandardizados e normativamente referenciados: Participação Social (SOC), Visão (VIS), Audição (HEA), Toque (TOU), Conhecimento do Corpo (BOD), Equilíbrio e Movimento (BAL), Planejamento de Ideias (PLA) e Total dos Sistemas Sensoriais (TSS). Esta escala avalia o processamento sensorial, a práxis e a participação social de crianças entre os 5 e os 12 anos de idade, que estejam frequentando a escola. As perguntas que compõe a avaliação são relacionadas ao comportamento que a criança apresenta em determinadas situações, referente a estímulos sensoriais apresentados. Assim, os pais respondem com que frequência a criança apresenta determinado comportamento frente ao estímulo sensorial, em uma escala Likert em que as opções são nunca, ocasionalmente, frequentemente e sempre (pontuando de 1 a 4 respectivamente). Ao final, as pontuações de cada escala (SOC; VIS; HEA; TOU; BOD; BAL; PLA e TSS) são somadas individualmente e colocadas em uma tabela para correspondência de escores T. Os escores variam de 40 a 80T, e a pontuação de cada escala classifica o funcionamento do processamento sensorial em três tipos de interpretação: Típico, Algum Problema ou Disfunção Definitiva, relacionada a cada sistema sensorial e a soma final de todos os sistemas avaliados. Assim, quanto maior for a pontuação da criança (maior seu escore T), maior será a alteração do seu processamento sensorial. Para o presente estudo, foram consideradas apenas as escalas correspondentes aos sistemas sensoriais (visual (VIS), auditivo (HEA), tátil (TOU), proprioceptivo (BOD) e vestibular (BAL)) e ao total dos sistemas (TSS). Os resultados “algum problema” e “disfunção definitiva” foram categorizados como “disfunção”, já que qualquer alteração em um dos sistemas já se caracteriza como um problema de processamento sensorial^(22)^.

A avaliação da motricidade orofacial foi realizada através do protocolo “Avaliação Miofuncional Orofacial com Escores” (AMIOFE). Todas as avaliações foram gravadas para posterior marcação pelo mesmo profissional fonoaudiólogo que realizou a avaliação do protocolo de sinais e sintomas da respiração oral. Esse instrumento tem como objetivo a avaliação miofuncional orofacial e é dividido em aspectos como aparência e condição postural/posição (lábios, mandíbulas, bochechas, face, língua e palato); mobilidade (lábios, língua, mandíbula e bochechas); funções (mastigação, deglutição e respiração) e outros comportamentos e sinais de alteração (movimentação da cabeça ou outras partes do corpo, postura alterada e escape de alimento). Cada um desses aspectos é avaliado através de observação e pontuado em escores, segundo Felício e Ferreira^(23)^.

### Procedimento

Inicialmente foi realizada a seleção da população do grupo caso (RO) através da observação dos prontuários disponibilizados pelo serviço onde as avaliações estavam sendo realizadas (Ambulatório de Alergia e Alergologia do Hospital das Clínicas da Universidade Federal de Pernambuco). Após a seleção das crianças pelos prontuários, através dos critérios de elegibilidade, no dia do atendimento agendado pelo serviço, foram descritos os procedimentos da pesquisa aos pais ou responsáveis pela criança para posterior consentimento. Quando do aceite em participar, após todos os envolvidos (responsáveis) assinarem o Termo de Consentimento Livre e Esclarecido, as crianças eram encaminhadas para realizar o diagnóstico de respiração oral, através do protocolo de identificação de sinais e sintomas da respiração oral. Após a identificação, era realizada uma entrevista e preenchida a ficha de registro de dados, para coleta dos dados sociodemográficos e alguns critérios de elegibilidade. Após essa triagem, caso não houvesse viabilidade, as avaliações eram agendadas para a data do próximo comparecimento da criança ao serviço.

Quando da possibilidade de ser realizada a avaliação logo após a triagem, eram aplicados os instrumentos de avaliação do processamento sensorial, em forma de entrevista, através da *SPM – Home Form*, e a motricidade orofacial, através do instrumento AMIOFE. Os protocolos de avaliação foram aplicados na presença dos pais ou responsáveis pela criança, em sala destinada a esse fim, no ambulatório onde estava sendo realizada a coleta dos dados.

Após a coleta da população do grupo caso (RO), o grupo controle (RN) foi selecionado por pareamento de idade e sexo. Cabe esclarecer que os participantes do grupo controle não foram coletados no mesmo local e tempo que o grupo caso, já que a seleção foi feita por pareamento de idade e gênero, sendo necessário finalizar a coleta do grupo caso para posterior seleção do grupo controle. Após o pareamento, os critérios de elegibilidade foram coletados através de observações dos prontuários fornecidos pela Clínica escola de Odontologia da Universidade Federal de Pernambuco e de entrevista inicial com os pais ou responsáveis pela criança. Após a triagem, quando do aceite em participar da pesquisa, as crianças eram encaminhadas para realizar o diagnóstico de respiração oral, através do protocolo de identificação de sinais e sintomas da respiração oral. Quando o resultado era de respiração nasal, dava-se a avalição do processamento sensorial através da *SPM – Home Form.*

Para a avaliação da motricidade orofacial, os participantes do grupo caso (RO) permaneceram em uma cadeira com encosto, em posição ereta; com os pés apoiados, os membros superiores e inferiores relaxados e descruzados; com as mãos sobre as coxas, a mandíbula em posição paralela ao solo; com a cabeça sem apoio. A escolha desta postura proporciona mais conforto e espontaneidade à cabeça e ao pescoço. Para avaliação da deglutição e mastigação, os participantes foram orientados a ingerir um copo de 180 ml de água mineral sem gás e um pão do tipo francês (25g) que foi pesado por uma balança de Precisão JL-3 (capacidade 500mg), de forma habitual, respectivamente^(23)^. Toda a aplicação deste protocolo era acompanhada e orientada por um fonoaudiólogo, e filmada com o consentimento dos pais ou responsáveis para posterior análise. A aplicação da SPM foi realizada por uma terapeuta ocupacional através de entrevista com os pais ou responsáveis pelas crianças.

### Análise dos dados

Para a análise dos dados, foi utilizado o Software SPSS (*Statistical Package for the Social Sciences*) na versão 18 e o Excel 2010^®^. Os resultados foram apresentados em forma de tabela e com suas respectivas frequências absoluta e relativa. Para verificar a existência de associação entre o processamento sensorial e as características clínicas estudadas, foi utilizado o Teste Qui-Quadrado ou o Teste Exato de Fisher, quando necessário. Todos os testes foram aplicados com 95% de intervalo de confiança e os valores de p < 0,05 foram considerados estatisticamente significativos.

Este projeto foi submetido ao Comitê de Ética em pesquisa com seres humanos do Centro de Ciências da Saúde da Universidade Federal de Pernambuco, de acordo com a Resolução 466/12 do Conselho Nacional de Saúde do Ministério da Saúde do Brasil referente a pesquisas com seres humanos. O mesmo foi aceito através do parecer nº 992.769, CAAE: 42103315.3.0000.5208. As avaliações só foram realizadas após todos os envolvidos (responsáveis) assinarem o Termo de Consentimento Livre e Esclarecido.

## RESULTADOS

Participaram do estudo 100 crianças, sendo 50 respiradoras orais (RO) e 50 respiradoras nasais (RN), 68 do sexo masculino e 32 do sexo feminino, com idade média de 8 (± 2,15) anos e frequentando o ensino fundamental I (64% RO; 84% RN). A maioria das mães (82%) apresentou idade média de 35,2 (± 7,2) anos e escolaridade de ensino médio completo (54,0% RO; 56% RN).

Ao analisar o processamento sensorial (Tabela 1) foi verificado que 41 (68,0%) crianças respiradoras orais apresentaram disfunção do processamento sensorial. Os sistemas sensoriais que apresentaram maior frequência de disfunção nessa população foram o visual (65,0%), o tátil (toque) (70,0%), e o vestibular (equilíbrio e movimento) (78,0%). Quando comparado ao processamento sensorial de crianças respiradoras nasais, a análise de todos os sistemas apresentou significância estatística, com mais atenção ao escore total (total dos sistemas sensoriais) e ao sistema vestibular (equilíbrio e movimento), ambos com valor de p = 0,000.

**Tabela 1:** Distribuição das frequências dos dados do processamento sensorial do grupo caso (RO) e controle (RN)

**Grupo Caso/Controle**	Sistema Sensorial **(Total Sistemas Sensoriais)**			p*	**OR**	**IC (95%)**
	Típico N (%)	Disfunção N (%)		<10^-4^	0,13	0,05-0,34
OB	9 (22%)	41 (68%)	
RN	31 (78%)	19 (32%)	
Total	40 (100%)	60 (100%)	
**Grupo Caso/Controle**	Sistema Sensorial **(Visual)**			p*	**OR**	**IC (95%)**
	Típico N (%)	Disfunção N (%)		0,004	0,31	0,14-0,71
RO	20 (37%)	30 (65%)				
RN	34 (63%)	16 (35%)				
Total	54 (100%)	46 (100%)				
**Grupo Caso/Controle**	Sistema Sensorial **(Visual)**			p*	**OR**	**IC (95%)**
	Típico N (%)	Disfunção N (%)		0,004	0,31	0,14-0,71
RO	20 (37%)	30 (65%)				
RN	34 (63%)	16 (35%)				
Total	54 (100%)	46 (100%)				
**Grupo Caso/Controle**	Sistema Sensorial **(Auditivo)**			p*	**OR**	**IC (95%)**
	Típico N (%)	Disfunção N (%)		0,028	0,40	0,17-0,95
RO	28 (65%)	22 (42%)				
RN	38 (58%)	12 (35%)				
Total	66 (100%)	34 (100%)				
**Grupo Caso/Controle**	Sistema Sensorial **(Tátil)**			p*	**OR**	**IC (95%)**
	Típico N (%)	Disfunção N (%)		0,001	0,23	0,10-0,55
RO	20 (35%)	30 (70%)				
RN	37 (65%)	13 (30%)				
Total	57 (100%)	43 (100%)				
**Grupo Caso/Controle**	Sistema Sensorial **(Proprioceptivo)**			p*	**OR**	**IC (95%)**
	Típico N (%)	Disfunção N (%)		0,008	0,34	0,15 – 0,77
RO	21 (38%)	29 (64%)				
RN	34 (62%)	16 (36%)				
Total	55 (100%)	45 (100%)				
**Grupo Caso/Controle**	Sistema Sensorial **(Vestibular)**			p*	**OR**	**IC (95%)**
	Típico N (%)	Disfunção N (%)		<10^-4^	0,14	0,060-0,37
RO	20 (33%)	30 (78%)				
RN	41 (67%)	9 (22%)				
Total	61 (100%)	39 (100%)				

Legenda: OR = Odds ratio; IC = Intervalo de confiança; p*>0,05

No que se relaciona aos aspectos miofuncionais de mobilidade, a maioria das crianças RO não apresentou alteração de movimentos labiais, da língua, da mandíbula e das bochechas (Tabela 2). Nas funções do Sistema Estomatognático (deglutição e mastigação), foi percebido a presença de movimentação da cabeça, tensão dos músculos faciais e escape do alimento para a maioria da população estudada (Tabela 2). Dentro da função da mastigação, o tipo de mastigação mais frequente foi a bilateral alternada (42,2%), seguida da unilateral preferencial (40,0%) e da unilateral crônica (18,0%).

**Tabela 2:** Frequência dos aspectos miofuncionais orofaciais de mobilidade labial, da língua, da mandíbula e das bochechas e das funções de deglutição e mastigação do grupo caso (RO), Recife-PE, 2017

**Variável (Mobilidade)**	**Normal**		**Alterado**	
	*F*	%	*f*	%
Movimentos Labiais	31	62,0	19	38,0
Movimentos da Língua	39	78,0	11	22,0
Movimentos da Mandíbula	36	72,0	14	28,0
Movimento das Bochechas	41	82,0	09	18,0
**Variável (Deglutição)**	**Ausente**		**Presente**	
	*F*	%	*f*	%
Movimentação da cabeça	19	38,0	31	62,0
Tensão dos Músculos Faciais	23	46,0	27	54,0
Escape de Alimentos	19	38,0	31	62,0
**Variável (Mastigação)**	**Ausente**		**Presente**	
	*F*	%	*f*	%
Movimentação da cabeça	20	40,0	30	60,0
Postura alterada	16	32,0	34	68,0
Escape de Alimentos	18	36,0	32	64,0

No que se refere a relação entre processamento sensorial e o Sistema Estomatognático, houve relação com significância estatística entre o processamento sensorial proprioceptivo (consciência do corpo) e o aspecto miofuncional de mobilidade da bochecha (p= 0,040) e entre o processamento visual e os movimentos de cabeça durante a deglutição (p=0,042). O processamento sensorial tátil e o tipo de mastigação também apresentaram significância estatística (p= 0,03). É possível perceber, através da regressão logística binária, que as crianças com respiração oral têm maior tendência de apresentar alteração de processamento sensorial e mais especificamente no processamento sensorial vestibular, ou seja, no equilíbrio e movimento (Tabela 3).

**Tabela 3:** Regressão Logística Binária mostrando a influência da combinação de todos os fatores da respiração oral no processamento sensorial de crianças, Recife-PE, 2017

**Sistema**	**B**	**P**	**OR**	**IC 95%**
TSS	1,61	0,030*	4,999	1,16-21,45
Visual	0,215	0,709	1,239	0,40-3,82
Auditivo	-0,569	0,365	,566	0,16-1,94
Tátil	0,343	0,577	1,410	0,42-4,71
Proprioceptivo	-0,315	0,609	,730	0,22-2,44
Vestibular	1,594	0,005*	4,923	1,61-15,07

Legenda: TSS = Total dos sistemas sensoriais; B = Coeficiente B; OR = Odds ratio; IC = Intervalo de confiança; p*>0,05

## DISCUSSÃO

Após análise dos dados foi possível perceber que o processamento sensorial de todos os sistemas apresenta alteração nos respiradores orais e que esse mau processamento se relaciona a mobilidade orofacial, bem como com as funções de deglutição e mastigação.

A população estudada foi semelhante à de outros estudos que avaliaram crianças respiradoras orais, sendo a maioria do sexo masculino e estando com média de idade de oito anos, estudando no ensino fundamental^(8,11,13,21,24)^. Cabe salientar que a faixa etária selecionada nesse estudo esteve limitada à especificidade do instrumento de avaliação do processamento sensorial, que contém perguntas relacionadas a faixa etária de 5 a 12 anos. É importante frisar também que a população de respiradores nasais foi pareada por sexo e idade com a população de respiradores orais, evitando qualquer especificidade quanto a essas variáveis. A idade materna e o grau de escolaridade dos responsáveis também favoreceram as respostas aos instrumentos, já que alguns deles dependiam da interpretação e respostas dentro de escala *Likert*.

Ao contrário do observado nas crianças respiradoras orais, não foram detectadas alterações no processamento sensorial das crianças respiradoras nasais. Isso nos faz perceber a influência da respiração oral neste processamento, já que essa condição não permite um adequado input sensorial, dificultando o registro das sensações e possivelmente a alteração de toda a sequência do processamento, levando a uma resposta adaptativa inadequada ao ambiente e dificuldade no desempenho de atividades cotidianas. É importante ressaltar que os sistemas sensoriais funcionam de forma integrada para o desempenho ótimo das ações humanas, desta forma, cada um deles depende do bom funcionamento do outro para que as respostas adaptativas às demandas ambientais sejam adequadas^(25)^.

As alterações encontradas nos sistemas visual, vestibular e proprioceptivo, na população de respiradores orais estudada, podem ser justificadas pela postura de cabeça, pescoço e ombro comum aos respiradores orais. A modificação da posição da cabeça e dos ombros exige que o respirador oral incline o corpo para frente, leve os braços para trás e os pés estejam em inversão, para conseguir equilibrar-se^(26)^. A harmonia do corpo com relação aos seus segmentos e ao ambiente que o rodeia depende da integração da informação advinda dos sistemas visual, somatossensorial e vestibular, mantendo o equilíbrio corporal eficiente e postura adequada^(25)^. Portanto, a disfunção de qualquer um desses sistemas pode afetar o controle postural e o equilíbrio. Dessa forma, os resultados obtidos concordam com a literatura já existente sugerindo a relação do processamento sensorial proprioceptivo e vestibular com o padrão de respiração oral das crianças devido às alterações de postura.

Em uma revisão de literatura, Machado et al.^(26)^ apresentaram artigos que relacionaram a postura corporal com as funções do Sistema Estomatognático, postulando que alterações nas funções estomatognáticas, principalmente a respiração, podem contribuir para deficiências no alinhamento e controle postural, devido a relação entre o sistema trigeminal e as estruturas nervosas envolvidas no controle da postura. Em um estudo observacional, com grupo controle, Conti et al.^(6)^ avaliaram 306 respiradores orais (RO) e 124 respiradores nasais (RN), observando uma alteração postural moderada em 60,74% na população de RO, com diferença estatisticamente significativa (p ≤ 0,0002) quando compararam a posição dos segmentos cabeça, ombros, pés e arco plantar do grupo caso com o grupo controle (RN). Roggia e colaboradores^(9)^ avaliaram os sistemas vestibular, visual e somatossensorial de forma integrada, apresentando a influência dos três sistemas no equilíbrio e postura de crianças respiradoras orais. Os autores concluíram que os respiradores orais apresentaram mais alterações posturais que o grupo controle (respiradores nasais) e relacionaram as dificuldades encontradas com às modificações estruturais e fisiológicas ocasionadas nos sistemas sensoriais.

Neste sentido, as respostas do processamento visual e tátil, apresentadas pelos respiradores orais desse estudo, além de se relacionarem aos aspectos posturais apresentados por essa população, também podem ser justificadas por outros fatores. Em relação ao processamento visual, a avaliação utilizada (SPM – *home form*) obtém os resultados através de questões relacionadas as respostas que os indivíduos oferecem frente a um estímulo sensorial. Assim, grande parte das perguntas da sessão que avalia o sistema visual estão direcionadas a atenção. Alguns estudos relatam que respiradores orais apresentam problemas de atenção e concentração, devido as demandas respiratórias que prejudicam, entre outros fatores, o descanso e sono, tornando essa população mais sonolenta durante o dia e consequentemente mais desatenta^(12,14)^.

Já o sistema tátil está diretamente relacionado ao sistema proprioceptivo e gustativo. Este último é diretamente influenciado pelo sistema olfativo e consequentemente à respiração. Sendo assim, os respiradores orais desse estudo, devido à diminuição do olfato, do paladar e de tônus da musculatura orofacial e a má oclusão oral (mordida aberta ou cruzada e ausência de elementos dentários anteriores), podem ter selecionado o alimento pela consistência e consequentemente facilidade na ingestão, optando por consumir alimentos pastosos e líquidos^(13,26)^. Essa restrição alimentar diminui a possibilidade de experiências sensoriais orais táteis e possivelmente corporais, levando a ausência de experimentação sensorial adequada, pobre registro das informações e mau processamento sensorial.

Do ponto de vista da Integração Sensorial, os sentidos proprioceptivo, vestibular e tátil são enfatizados por serem primitivos e primários, dominando a interação das crianças com o mundo nos primeiros anos de vida^(10)^. Assim, devido às posturas corporais assumidas e consequentemente as alterações de processamento vestibular, tátil, proprioceptivo e visual, as crianças respiradoras orais avaliadas podem ter apresentado características de funções motoras desorganizadas, tônus muscular diminuído e equilíbrio deficitário. Essas funções estão relacionadas a práxis, uma habilidade que depende do bom funcionamento e integração dos sistemas sensoriais, especialmente tátil, proprioceptivo e vestibular para conceituar, planejar e executar um ato motor não habitual. Desta forma, alterações nesses sistemas podem levar a dificuldade na realização de atividades motoras, como a transição de uma posição do corpo para outra e ações sequenciadas ou cronometradas envolvidas em uma tarefa. Estas características atrapalham o desempenho das crianças em atividades diárias como alimentação, vestir-se, escrever e atividades escolares, representando um déficit de desempenho significativo que pode acarretar problemas de participação social, considerando essa fase do desenvolvimento da criança, onde estão sendo iniciadas as formações e identificações grupais^(10,18,19)^.

No que se refere ao sistema auditivo, foi observado mais respiradores orais com processamento sensorial típico (56%). Porém, quando comparados ao grupo controle (76% de processamento típico) é possível observar uma diferença significativa, o que reflete uma influência da respiração oral no processamento destas informações, já que as crianças foram pareadas por idade e gênero, e encontram-se em processo educacional semelhante. Uma das consequências da respiração oral, devido ao mau funcionamento da tuba auditiva, é o desenvolvimento de otite média e, consequentemente, de flutuação da audição^(11,27)^. Portanto, a respiração oral é uma condição que pode estar associada a uma falha no processamento auditivo, sendo otites e flutuação da audição possíveis causas desse problema. Em crianças em idade escolar, como no caso dessa pesquisa (5 – 12 anos), os efeitos da respiração oral no sistema auditivo merecem uma atenção diferenciada, uma vez que é nessa época que há o maior desenvolvimento das habilidades auditivas do ser humano e, portanto, flutuações da audição devem ser observadas para que ocorra o desenvolvimento normal desse sistema, evitando dificuldades de aprendizagem^(28)^.

Em estudo com população semelhante, Correa et al.^(27)^, encontraram resultados significativos entre os grupos caso (respiradores orais) e controle (respiradores nasais) para as condições de orelhas esquerda e direita competitiva, alteração relacionada a organização e integração auditiva, fechamento auditivo e padrão temporal, concluindo que as crianças respiradoras orais apresentam menor desempenho nas habilidades do processamento auditivo do que as crianças respiradoras nasais. Cabe salientar que a avaliação do processamento sensorial realizada no presente estudo observa as respostas comportamentais adaptativas aos estímulos recebidos, avaliando de que forma as crianças reagem na maior parte do tempo as demandas do ambiente. Essa análise se diferencia de grande parte dos estudos relacionados ao processamento auditivo, que se detém a quantificação dos impulsos elétricos, bem como a presença ou ausência de respostas auditivas e da fala^(11,27,29)^.

Independente da causa, alterações no processamento sensorial auditivo podem influenciar no desempenho dessas crianças, especialmente nas atividades escolares, já que essa disfunção pode levar a dificuldades no desenvolvimento da linguagem, déficits de atenção, erros gráficos e de leitura, lentidão, dificuldade para seguir instruções orais e para selecionar estímulos auditivos em ambientes ruidosos, agitação, hiperatividade ou apatia e alterações da noção de lateralidade^(24,27,28)^.

Em relação ao Sistema Estomatognático, não foram encontradas, na maioria da população de respiradores orais, alterações na mobilidade de lábios, língua, mandíbula e bochecha. Porém, nas funções de deglutição e mastigação foi observada presença de movimentação da cabeça, tensão dos músculos faciais e escape do alimento. Essas alterações mostram uma inabilidade nessas funções, provavelmente por diminuição da contração de alguns músculos, contração da musculatura periorbicular e flacidez dos órgãos fonoarticulatórios. Essas características também estiveram presentes em estudos que compararam crianças respiradoras orais e nasais^(13,26)^.

Para Machado et al.^(26)^. o RO, em muitos casos, não pode mastigar corretamente o alimento devido à necessidade de respirar, pois ao abrir a boca para tal, há adaptações e desequilíbrio das estruturas e funções orofaciais que comprometem a mastigação e a deglutição, e, consequentemente, gera dificuldades na alimentação. A criança respiradora oral deve manter livre a passagem de ar pela boca para que consiga respirar. Assim, ao ingerir o alimento, tem necessidade de degluti-lo rapidamente para liberar a passagem de ar pela boca para voltar a respirar^(13)^. Nesse sentido, a literatura mostra que o tempo da mastigação do RO é mais rápido do que o do indivíduo que respira pelo nariz^(13,26,29)^, o que pode justificar as alterações encontradas nas funções de mastigação e deglutição.

Quanto ao tipo de mastigação, a maior parte apresentou mastigação bilateral alternada (42%), padrão que é considerado como o ideal e responsável pela existência de um equilíbrio orofacial. Esse resultado foi semelhante ao estudo de Silva et al.^(29)^. que identificou, numa amostragem menor, a prevalência da mastigação bilateral alternada em 20 das 23 crianças RO avaliadas. Isso pode ser justificado pelo fato de que a respiração oral pode trazer alteração nas estruturas do Sistema Estomatognático dependendo do grau de severidade da obstrução nasal e do tempo de interferência, sendo subjetiva essa avaliação por se tratar de uma população ainda em desenvolvimento. Apesar disso, houve uma aproximação da porcentagem de RO com mastigação unilateral preferencial (40%), o que aproxima os resultados aos de estudo realizados com população semelhante, na mesma faixa etária^(13,29)^.

Quando o processamento sensorial foi relacionado com as estruturas e funções do Sistema Estomatognático de respiradores orais, foi possível observar resposta estatisticamente significativa entre o processamento proprioceptivo e a movimentação de bochecha; processamento visual e movimentação de cabeça durante a deglutição e a relação do tipo de mastigação com o processamento tátil. Esses dados mostram a possível influência da respiração oral no mau processamento das informações sensoriais e as consequências que isto pode trazer as funções estomatognáticas. No que se refere ao processamento proprioceptivo e a movimentação de bochecha, é possível observar que apesar da disfunção proprioceptiva, a maioria dos respiradores orais apresentou movimentação normal de bochecha, o que pode ser justificado pelo tipo de material (pão e água) que foram utilizados para avaliar a mastigação e deglutição. Esses suprimentos, provavelmente, não exigem inputs proprioceptivos diferentes dos habituais para essa população, sendo assim, mesmo com o mau processamento proprioceptivo, a movimentação não sofreu alteração, por não exigir mais desse sistema.

As respostas obtidas pela relação entre o processamento visual e a movimentação de cabeça durante a deglutição mostra que a maior disfunção no processamento visual ocorreu quando houve presença de movimentação da cabeça durante a deglutição. Sabe-se que o respirador oral assume postura de compensação da cabeça para manter o plano bipupilar da linha do horizonte paralelo ao solo para a manutenção do equilíbrio, isso ocorre através de uma pequena inclinação da cabeça para trás^(8,9)^. Este fato pode justificar a dificuldade de manutenção visual durante a deglutição e presença da movimentação da cabeça nesta função. Outro ponto a ser considerado é a grande quantidade de estímulos sensoriais presentes na atividade da alimentação e na função da deglutição, o que pode requerer maior divisão de atenção dos estímulos, fator este prejudicado em crianças respiradoras orais, principalmente na relação com o processamento visual.

No que tange a relação entre o tipo mastigatório e o processamento tátil, observa-se que quando o processamento é típico a mastigação apresenta-se alternada, já quando há disfunção do processamento a mastigação ocorre com preferência. Esses dados apresentam a influência que o processamento tátil (principalmente o input sensorial) pode exercer na mastigação e no tipo mastigatório. Sabendo-se que os respiradores orais apresentam seletividade alimentar, principalmente relacionada a textura e consistência dos alimentos, a falta de estimulação e exploração nessa região justifica a disfunção tátil apresentada e o tipo de preferência mastigatória. A mastigação passa por modificações até o amadurecimento do Sistema Estomatognático e o desenvolvimento total da dentição. A partir daí, ocorre o aperfeiçoamento da mesma e, paralelamente, uma adaptação morfológica da superfície oclusal. A musculatura mastigatória tem o papel mais importante no processo, apesar de participarem também os músculos da língua e os músculos faciais^(13,29)^. Diante disso, a consistência do alimento influencia diretamente na mastigação, adaptando-a ao tipo de alimento ingerido, modificando a intensidade da força, a pressão e a quantidade de golpes mastigatórios, estimulando e trabalhando a musculatura mastigatória. Assim, a consistência do alimento irá influenciar tanto o tipo de estímulo tátil fornecido, quanto a preferência do lado mastigatório.

Apesar de não ter sido encontrada relação estatística significante entre os outros sistemas sensoriais e as funções estomatognáticas, é possível perceber que há uma tendência a presença de movimentação da cabeça, tensão dos músculos faciais e escape do alimento durante a mastigação e a deglutição quando o processamento sensorial está disfuncional. Esses dados mostram que as alterações apresentadas nas funções de mastigação e deglutição dos respiradores orais avaliados podem estar sendo influenciadas pela alteração no processamento sensorial, já que este deve ocorrer de forma integrada para garantir um desempenho adequado nas atividades cotidianas^(25)^. Quando há qualquer alteração de um desses sistemas, ocorre uma relação de feedback negativo com os outros sentidos, ocasionando déficits na realização das atividades.

Por ter o objetivo de apresentar a ausência ou a presença de alteração no processamento sensorial de respiradores orais, algumas inferências não puderam ser realizadas neste estudo, já que necessitariam de uma classificação do tipo de alteração do processamento sensorial de cada sistema. Outro ponto a ser considerado foi o estudo realizado com população de apenas uma cidade do Brasil, o que pode ter influenciado nas respostas dadas, principalmente as do *SPM Home Form*, que por ser um instrumento que avalia as respostas adaptativas ao ambiente sofre influência da interpretação e da cultura populacional. Assim, sugerem-se estudos que avaliem população de diferentes estados e regiões brasileiras, bem como que caracterizem o tipo de transtorno do processamento sensorial de cada sistema avaliado, garantindo assim a possibilidade de inferências sobre a quantidade de estímulos oferecidos e o direcionamento das práticas clínicas.

## CONCLUSÃO

Desta forma, os achados mostraram que o processamento sensorial de crianças respiradoras orais se encontra alterado para todos os sistemas avaliados, principalmente quando comparado com os dados das crianças respiradoras nasais. Apresenta também que houve relação significativa entre o processamento sensorial proprioceptivo, visual e tátil com a movimentação das bochechas e da cabeça durante a deglutição, bem como com o tipo de mastigação realizada pelos respiradores orais.

Por apresentar apenas a presença ou ausência de alterações no processamento sensorial, este estudo sugere aprofundamento nas observações clínicas dessa população para caracterizar o tipo de transtorno do processamento sensorial de cada sistema e garantir uma intervenção direcionada e individualizada, considerando as características pessoais das atividades desempenhadas e dos contextos de vida. Por fim, essas observações oferecem base para um olhar mais ampliado quanto a população de respiradores orais, no que se refere a interação dos sistemas sensoriais com essa condição de saúde e a melhor tomada de decisão para intervenções multiprofissionais, incluindo os terapeutas ocupacionais na avaliação e intervenção através da terapia de Integração Sensorial, tipo de tratamento realizado apenas por este profissional.
